# Supplementary material for: A fault-intrusion-tolerant system and deadline-aware algorithm for scheduling scientific workflow in the cloud
Source: PeerJ Comput Sci. 2021 Nov 2;7:e747. doi: 10.7717/peerj-cs.747 (PMC8576554; doi:10.7717/peerj-cs.747)
Supplement: Supplemental Information 6 [file peerj-cs-07-747-s006.zip › Myproject1.1/docs/org/cloudbus/cloudsim/network/GraphReaderBrite.html]

GraphReaderBrite


JavaScript is disabled on your browser.


- Overview
- Package
- Class
- Use
- Tree
- Deprecated
- Index
- Help

- Prev Class
- Next Class

- Frames
- No Frames

- All Classes

- Summary:
- Nested |
- Field |
- Constr |
- Method

- Detail:
- Field |
- Constr |
- Method


org.cloudbus.cloudsim.network

## Class GraphReaderBrite

- java.lang.Object
- - org.cloudbus.cloudsim.network.GraphReaderBrite

- All Implemented Interfaces:
  :   GraphReaderIF

  ---

    

  ```
  public class GraphReaderBrite
  extends java.lang.Object
  implements GraphReaderIF
  ```

  This class is just an file-reader for the special brite-format! the brite-file is structured as
  followed: Node-section: NodeID, xpos, ypos, indegree, outdegree, ASid, type(router/AS)
  Edge-section: EdgeID, fromNode, toNode, euclideanLength, linkDelay, linkBandwith, AS\_from, AS\_to,
  type

  Since:
  :   CloudSim Toolkit 1.0

- - ### Constructor Summary

    Constructors

    Constructor and Description

    `GraphReaderBrite()`
  - ### Method Summary

    Methods

    | Modifier and Type | Method and Description |
    | --- | --- |
    | `TopologicalGraph` | `readGraphFile(java.lang.String filename)` this method just reads the file and creates an TopologicalGraph object |

    - ### Methods inherited from class java.lang.Object

      `clone, equals, finalize, getClass, hashCode, notify, notifyAll, toString, wait, wait, wait`

- - ### Constructor Detail


    - #### GraphReaderBrite

      ```
      public GraphReaderBrite()
      ```
  - ### Method Detail


    - #### readGraphFile

      ```
      public TopologicalGraph readGraphFile(java.lang.String filename)
                                     throws java.io.IOException
      ```

      this method just reads the file and creates an TopologicalGraph object

      **Specified by:**
      :   `readGraphFile` in interface `GraphReaderIF`

      Parameters:
      :   `filename` - name of the file to read

      Returns:
      :   created TopologicalGraph

      Throws:
      :   `java.io.IOException`


- Overview
- Package
- Class
- Use
- Tree
- Deprecated
- Index
- Help

- Prev Class
- Next Class

- Frames
- No Frames

- All Classes

- Summary:
- Nested |
- Field |
- Constr |
- Method

- Detail:
- Field |
- Constr |
- Method
